# Supplementary material for: Integrating molecular, histopathological, neuroimaging and clinical neuroscience data with NeuroPM-box
Source: Commun Biol. 2021 May 21;4:614. doi: 10.1038/s42003-021-02133-x (PMC8140107; doi:10.1038/s42003-021-02133-x)
Supplement: Supplementary file 2 — Supplementary Material [file 42003_2021_2133_MOESM2_ESM.pdf]

SUPPLEMENTARY INFORMATION (one table and two figures)

Table S1. Main demographic characteristics for the two populations.

Variable	HBTRC<br>(N=736)	ADNI<br>(N=911)
Women	354 (47.9%)	424 (46.5%)
Age (years)	70.8 (15.10)	72.9 (7.21)
Education (years)	-	16.3 (2.69)
HC	173 (23%)	266 (29.2%)
Diseased (MCI, AD)	563 (77%)	645 (70.8%)

Data are number (%) or mean (std).

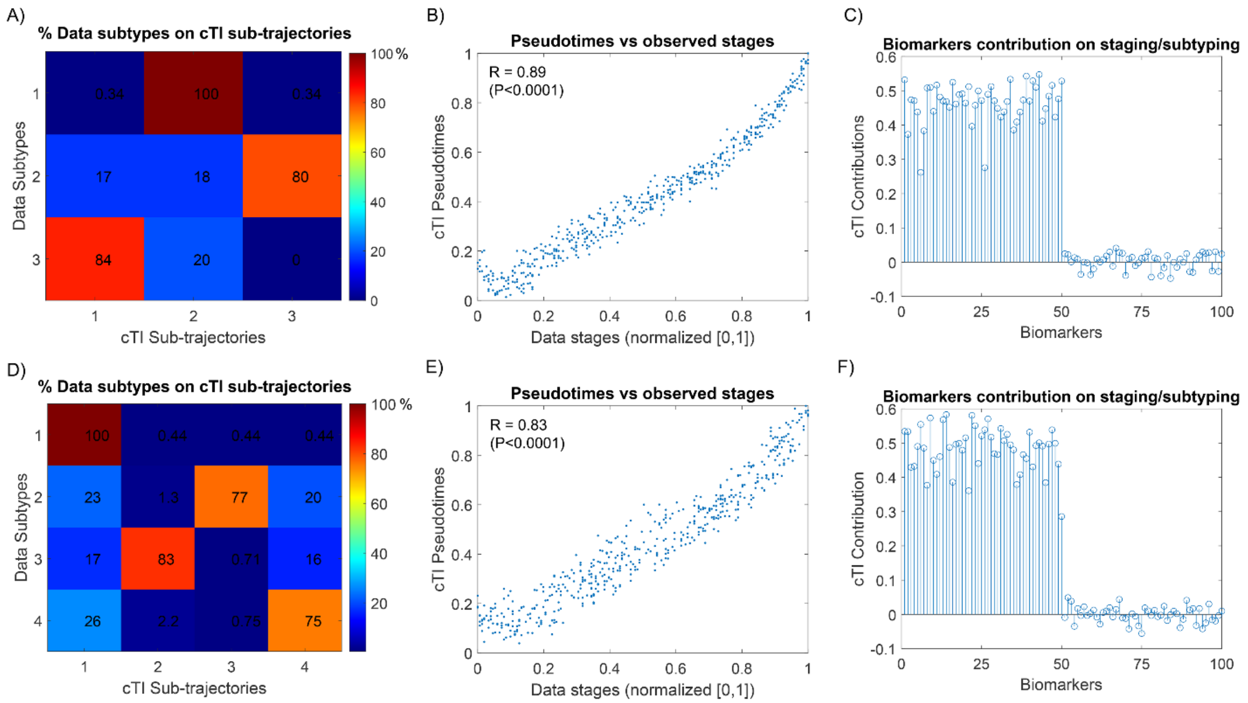

Fig. S1. cTI results with synthetic data. To test and illustrate the effect of different population characteristics on the cTI approach's performance (e.g. population heterogeneity, number of subjects), we simulated three datasets (see *Example Dataset 3*, *online Methods* section; synthetic data and demo script are available at the *NeuroPM-box*'s downloading page). We set the number of subtypes to be three (results in A-C, with two ‘diseased’ subpopulations and a control subgroup) and four (results in D-F, with three ‘diseased’ subpopulations and a control subgroup). The number of subjects for each case was set to be 500 (results in A-F) or 1000 subjects (results included only in the demo). To each dataset, the number of informative

biomarkers was set to be 50, adding other 50 randomly distributed biomarkers, for a total of 100 features. A) Correspondence (in %) between the subjects' original subtypes and the cTI's identified sub-trajectories, for the case of three subtypes. Notice that all the subtypes are recovered from an 80 to 100%. B) Relationship between simulated/observed disease stages and estimated cTI pseudotimes, for the two 'diseased' subtypes (each point corresponds to a subject). C) Obtained features contribution on the cTI's staging/subtyping, for the case of three subtypes. D) Correspondence between the subjects' original subtypes and the cTI's identified sub-trajectories, for the case of four subtypes. Notice that all the subtypes are recovered from a 75 to 100%. E) Estimated cTI pseudotimes vs original disease stages, for the three 'diseased' subtypes (each point corresponds to a subject). F) Features contribution on the cTI's staging/subtyping, for the case of four subtypes. In C and F, notice that the first 50 features (those simulated to be informative) present considerably higher contributions than the randomly distributed features.

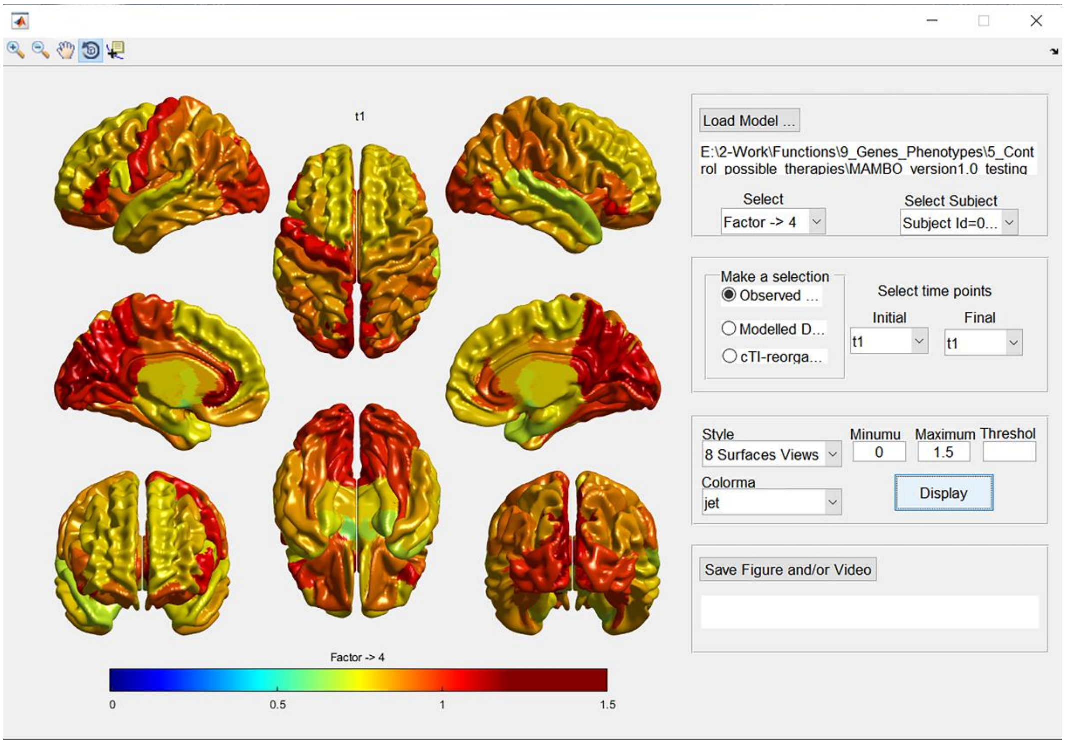

Fig. S2. *NeuroPM-viewer* interface depicting glucose metabolism (FDG PET) in an 8-brain surface view. In addition to multiple visualization settings (surface views, data-type, time windows, colormaps, minimum and maximum values, thresholds), the user can also save the depicted figures in traditional images formats (.jpg, .png, .tif) or as dynamic brain videos (.avi).
