# Supplementary material for: Integrating molecular, histopathological, neuroimaging and clinical neuroscience data with NeuroPM-box
Source: Commun Biol. 2021 May 21;4:614. doi: 10.1038/s42003-021-02133-x (PMC8140107; doi:10.1038/s42003-021-02133-x)
Supplement: Supplementary file 3 — Description of Additional Supplementary Files [file 42003_2021_2133_MOESM3_ESM.pdf]

Description of Additional Supplementary Files

File name: Supplementary data 1
Description: Source data underlying Figures 2e, f.

File name: Supplementary data 2
Description: Source data underlying Figures 3c, d.
