# Supplementary material for: Integrating molecular, histopathological, neuroimaging and clinical neuroscience data with NeuroPM-box
Source: Commun Biol. 2021 May 21;4:614. doi: 10.1038/s42003-021-02133-x (PMC8140107; doi:10.1038/s42003-021-02133-x)
Supplement: Supplementary file 6 — Reporting Summary [file 42003_2021_2133_MOESM6_ESM.pdf]

nature research

Corresponding author(s): Yasser Iturria-Medina

Last updated by author(s): Apr 6, 2021

Reporting Summary

Nature Research wishes to improve the reproducibility of the work that we publish. This form provides structure for consistency and transparency in reporting. For further information on Nature Research policies, see our Editorial Policies and the Editorial Policy Checklist.

Statistics

For all statistical analyses, confirm that the following items are present in the figure legend, table legend, main text, or Methods section.

n/a	Confirmed	
☐	☒	The exact sample size (n) for each experimental group/condition, given as a discrete number and unit of measurement
☐	☒	A statement on whether measurements were taken from distinct samples or whether the same sample was measured repeatedly
☐	☒	The statistical test(s) used AND whether they are one- or two-sided<br>*Only common tests should be described solely by name; describe more complex techniques in the Methods section.*
☐	☒	A description of all covariates tested
☐	☒	A description of any assumptions or corrections, such as tests of normality and adjustment for multiple comparisons
☐	☒	A full description of the statistical parameters including central tendency (e.g. means) or other basic estimates (e.g. regression coefficient) AND variation (e.g. standard deviation) or associated estimates of uncertainty (e.g. confidence intervals)
☐	☒	For null hypothesis testing, the test statistic (e.g. F, t, r) with confidence intervals, effect sizes, degrees of freedom and P value noted<br>*Give P values as exact values whenever suitable.*
☒	☐	For Bayesian analysis, information on the choice of priors and Markov chain Monte Carlo settings
☒	☐	For hierarchical and complex designs, identification of the appropriate level for tests and full reporting of outcomes
☒	☐	Estimates of effect sizes (e.g. Cohen's d, Pearson's r), indicating how they were calculated

Our web collection on statistics for biologists contains articles on many of the points above.

Software and code

Policy information about availability of computer code

Data collection	No software was used for data collection.
Data analysis	We used our freely available user-friendly software (NeuroPM-box, https://www.neuropm-lab.com/neuropm-box.html), which is provided as an easy-to-use standalone application for the major computer systems (macOS, linux, Windows) and includes a detailed tutorial. All the results presented in our study were obtained with this software. Detailed instructions about how to download and install it are provided in the manuscript. Additionally, a demo script for testing/evaluation is provided, including a user guide to reproduce all results presented in Fig. S1.

For manuscripts utilizing custom algorithms or software that are central to the research but not yet described in published literature, software must be made available to editors and reviewers. We strongly encourage code deposition in a community repository (e.g. GitHub). See the Nature Research guidelines for submitting code & software for further information.

Data

Policy information about availability of data

All manuscripts must include a data availability statement. This statement should provide the following information, where applicable:
- Accession codes, unique identifiers, or web links for publicly available datasets
- A list of figures that have associated raw data
- A description of any restrictions on data availability

All synthetic data used are available at the software's downloading page. All real data used are publicly available at the Gene Expression Omnibus (GEO; www.ncbi.nlm.nih.gov/geo, accession number GSE44772) and the Alzheimer's Disease Neuroimaging Initiative (ADNI; www.adni.loni.usc.edu). For reproducibility purposes, anonymized IDs of the studied ADNI subjects will be provided upon request, as well as detailed information of the imaging modalities and time points analyzed for each subject.

Field-specific reporting

Please select the one below that is the best fit for your research. If you are not sure, read the appropriate sections before making your selection.

- [x] Life sciences
- [ ] Behavioural & social sciences
- [ ] Ecological, evolutionary & environmental sciences

For a reference copy of the document with all sections, see nature.com/documents/nr-reporting-summary-flat.pdf

Life sciences study design

All studies must disclose on these points even when the disclosure is negative.

Sample size	Three different data populations were used (total N>4,547), including in-vivo (N=911) and post-mortem (N=736) neurodegenerative individuals, and synthetic subjects (N>2900).
Data exclusions	NA.
Replication	We performed five independent analysis on different datasets to validate the reproducibility of the contrastive Trajectories Inference (cTI) method, obtaining highly consistent results for the different scenarios/data. The datasets included Harvard Brain Tissue Resource Center [HBTRC] gene expression, HBTRC histopathology, and three synthetic datasets.
Randomization	For the population-based contrastive Trajectories Analysis (cTI), the gene expression and histopathological data were adjusted for post-mortem interval in hours (PMI), sample pH, RNA integrity number, age and gender.<br>Neuroimaging data was not adjusted, because the corresponding analyses were performed at the individual level.
Blinding	Blinding was not relevant to our study. All our analyses were unsupervised, i.e. not requiring any a priori training or fitting on cognitive or behavioural variables.

Reporting for specific materials, systems and methods

We require information from authors about some types of materials, experimental systems and methods used in many studies. Here, indicate whether each material, system or method listed is relevant to your study. If you are not sure if a list item applies to your research, read the appropriate section before selecting a response.

Materials & experimental systems

n/a	Involved in the study	
x		Antibodies
x		Eukaryotic cell lines
x		Palaeontology and archaeology
x		Animals and other organisms
	x	Human research participants
	x	Clinical data
x		Dual use research of concern

Methods

n/a	Involved in the study	
x		ChIP-seq
x		Flow cytometry
	x	MRI-based neuroimaging

Human research participants

Policy information about studies involving human research participants

Population characteristics	The human data (N=1,647) included in-vivo (911) and post-mortem (736) samples, with mean age 71(std 9), 47% women, and including healthy controls, Alzheimer's and Huntington's disease patients.
Recruitment	Subjects were recruited if were clinically diagnosed at baseline as healthy control (HC), early mild cognitive impairment (MCI), probable Alzheimer's disease patient, or Huntington's disease patient.
Ethics oversight	The ADNI data was collected according to Good Clinical Practice guidelines, the Declaration of Helsinki, US 21CFR Part 50 – Protection of Human Subjects, and Part 56 – Institutional Review Boards, and pursuant to state and federal HIPAA regulations (adni.loni.usc.edu). Study subjects (Table S1) and/or authorized representatives gave written informed consent at the time of enrollment for sample collection and completed questionnaires approved by each participating site Institutional Review Board (IRB). The authors obtained approval from the ADNI Data Sharing and Publications Committee for data use and publication, see documents http://adni.loni.usc.edu/wp-content/uploads/how_to_apply/ADNI_Data_Use_Agreement.pdf and http://adni.loni.usc.edu/wp-content/uploads/how_to_apply/ADNI_Manuscript_Citations.pdf, respectively.<br>All autopsied data were collected by the Harvard Brain Tissue Resource Center (HBTRC; GEO accession number GSE44772), and include subjects for whom both the donor and the next of kin had completed the HBTRC informed consent (http://www.brainbank.mclean.org/). Correspondingly, tissue collection and the research were conducted according to the HBTRC guidelines (http://www.brainbank.mclean.org/).

Note that full information on the approval of the study protocol must also be provided in the manuscript.

Clinical data

Policy information about clinical studies

All manuscripts should comply with the ICMJEguidelines for publication of clinical research and a completedCONSORT checklist must be included with all submissions.

Clinical trial registration	*Provide the trial registration number from ClinicalTrials.gov or an equivalent agency.*
Study protocol	*Note where the full trial protocol can be accessed OR if not available, explain why.*
Data collection	*Describe the settings and locales of data collection, noting the time periods of recruitment and data collection.*
Outcomes	*Describe how you pre-defined primary and secondary outcome measures and how you assessed these measures.*

Magnetic resonance imaging

Experimental design

Design type	Resting state.
Design specifications	NA.
Behavioral performance measures	NA.

Acquisition

Imaging type(s)	Resting state fMRI (r-fMRI), Arterial Spin Labeling (ASL), structural T1-MRI, and High angular resolution diffusion imaging (HARDI).
Field strength	3
Sequence & imaging parameters	r-fmri: echo-planar imaging sequence on a 3.0-Tesla Philips MRI scanner. Acquisition parameters were: 140 time points, repetition time (TR) = 3000 ms, echo time (TE) = 30 ms, flip angle = 80°, number of slices = 48, slice thickness = 3.3 mm, spatial resolution = 3×3×3 mm3 and in plane matrix = 64×64.<br>ASL: TR/TE = 3400/12 ms, TI1/TI2 = 700/1900 ms, FOV = 256 mm, 24 sequential 4 mm thick slices with a 25% gap between the adjacent slices, partial Fourier factor = 6/8, bandwidth = 2368 Hz/pix, and imaging matrix = 64 × 64.<br>HARDI (diffusion MRI): For each diffusion scan, 46 separate images were acquired, with 5 b0 images (no diffusion sensitization) and 41 diffusion-weighted images (b = 1000 s/mm2). Other acquisition parameters were: 256 × 256 matrix, voxel size: 2.7 × 2.7 × 2.7 mm3, TR = 9000 ms, 52 contiguous axial slices, and scan time 9 min.
Area of acquisition	Whole brain scans.

Diffusion MRI ☒ Used ☐ Not used

Parameters: 41 diffusion-weighted images (b = 1000 s/mm2) and 5 b0 images (no diffusion sensitization). No cardiac gating was used.

Preprocessing

Preprocessing software	SPM12 (www.fil.ion.ucl.ac.uk/spm).
Normalization	All structural T1 images underwent non-uniformity correction using the N3 algorithm (Sled et al., 1998). Next, they were segmented into grey matter, white matter and cerebrospinal fluid (CSF) probabilistic maps, using SPM12 (www.fil.ion.ucl.ac.uk/spm). Grey matter segmentations were standardized to MNI space (Evans et al., 1994) using the DARTEL tool (Ashburner, 2007). Each map was modulated in order to preserve the total amount of signal/tissue. Mean grey matter density and determinant of the Jacobian (DJ) (Ashburner, 2007) values were calculated for 78 regions covering all the brain's grey matter (Klein and Tourville, 2012). All the results/figures presented in this study correspond to the grey matter density.<br>All other imaging modalities were registered to the native T1 and from there normalized at the ICBM12 space by applying the T1's normalization transformation parameters. For further details, please see Methods.
Normalization template	ICBM152.
Noise and artifact removal	Motion correction across scans was performed in the resting state fMRI, using SPM12 (www.fil.ion.ucl.ac.uk/spm).
Volume censoring	NA.

Statistical modeling & inference

Model type and settings	NA.
Effect(s) tested	NA.

Specify type of analysis: ☐ Whole brain ☒ ROI-based ☐ Both

Anatomical location(s)	78 anatomically-defined regions covering all the brain's grey matter, proposed by Klein A, Tourville J. 101 Labeled Brain Images and a Consistent Human Cortical Labeling Protocol. Front. Neurosci. 2012; 6: 171.
Statistic type for inference (See Eklund et al. 2016)	NA.
Correction	NA.

Models & analysis

n/a	Involved in the study
☐	☐ Functional and/or effective connectivity
☐	☐ Graph analysis
☐	☒ Multivariate modeling or predictive analysis

Functional and/or effective connectivity	*Report the measures of dependence used and the model details (e.g. Pearson correlation, partial correlation, mutual information).*
Graph analysis	*Report the dependent variable and connectivity measure, specifying weighted graph or binarized graph, subject- or group-level, and the global and/or node summaries used (e.g. clustering coefficient, efficiency, etc.).*
Multivariate modeling and predictive analysis	Four different methods were implemented and extensively tested: contrasted Trajectories Inference (cTI), Multifactorial causal Model of Brain (Dis)organization (MCM), Epidemic Spreading Model (ESM), and personalized Therapeutic Intervention Fingerprint (pTIF). For detailed descriptions, please see corresponding mathematical definitions at the manuscript's Methods.
